# Prescription of Aspirin for adults with Diabetes

**DOI:** 10.4103/0973-3930.43099

**Published:** 2008

**Authors:** P. Sabitha, Asha Kamath, Prabha M. Adhikari

**Affiliations:** Department of Pharmacology, Kasturba Medical College, Mangalore, India; 1 Department of Community Medicine, Kasturba Medical College, Mangalore, India; 2 Department of Medicine, Kasturba Medical College, Mangalore, India

**Keywords:** Aspirin, cardio-vascular disease, diabetes mellitus.

## Abstract

**BACKGROUND:**

To evaluate the prescription of aspirin for primary and secondary prevention of cardiovascular disorders in diabetic patients, in the light of American Diabetes Association guidelines.

**MATERIALS AND METHODS:**

In this retrospective analysis, presence of any cardiovascular disease or cardiovascular disease risk factor as defined in American Diabetes Association guidelines and the use of aspirin and other medication data were extracted from the case files of 100 patients with type 2 diabetes mellitus visiting two teaching hospitals.

**RESULTS:**

Of 100 patients studied, 58% were men and 42% women and all were ≥ 40 years of age. 45% had at least one cardiovascular disease and all (100%) were on aspirin for secondary prevention; 45% had one or more risk factors, of which 11% (05/45) had aspirin prescribed for primary prevention; remaining 10% had neither risk factors nor cardiovascular disease (but age ≥ 40 years) and no aspirin documentation. Reasons for not using aspirin/antiplatelet drug were not recorded.

**CONCLUSIONS:**

American Diabetes Association recommendations for aspirin use for secondary prevention of cardiovascular diseases were strictly adhered to, in contrast to that for primary prevention. Under-prescription of aspirin could be attributed to the physicians' concern about the burden of poly-pharmacy and toxic effects of aspirin on long-term use. Extensive efforts are necessary to enhance aspirin use in this regard.

## Introduction

The risk of cardiovascular disease (CVD) and the resultant mortality is higher among diabetic patients than those without diabetes mellitus (DM).[1–2] American Diabetes Association (ADA) recommends aspirin therapy for the secondary and primary prevention/prophylaxis (SP and PP) of cardiovascular events in DM patients with established CVD or those with cardiovascular disease risk factors (CVDRFs), respectively.[3] In this retrospective analyses, we evaluated the documentation of aspirin use in diabetic patients attending two teaching hospitals.[3]

## Materials and Methods

Case files of 100 patients of type 2 DM, who were inpatients during the year 2005 at the two tertiary care hospitals attached to a teaching institution, were selected (first 50 names from each hospital data sheet) after obtaining permission from the concerned hospital authorities. Patient demographic details, CVDs, CVDRFs, and medications were recorded. We considered CVD as the presence of one or more of the five conditions (history of myocardial infarction (MI), vascular bypass procedure, stroke/transient ischemic attack, peripheral vascular disease (PVD), and angina), and CVDRFs as the presence of one or more of the five conditions (family history of CVD, hypertension, smoking, dyslipidemia, and albuminuria) as defined in ADA recommendation guidelines.[3] Age ≥ 40 years was considered as a separate CVDRF.[3] We recorded disease diagnoses as it was entered in the case files. Patient compliance was assumed if aspirin prescription was found documented in the case files. We estimated the percentage of diabetic patients with established CVD conditions and of those presenting with CVDRFs alone. The percentage of aspirin users in both the groups was analyzed.

## Results

Case files of 100 patients of type 2 DM were studied. The age of the patients ranged between 41 and 75 years, and 58% were men and 42% women. Table 1 gives the risk category and percent of aspirin users. Forty five percent had at least one of the CVDs, 45% had one or more CVDRFs alone. Remaining 10% had neither CVD nor any of the CVDRFs. Of the total, 50% of patients were prescribed aspirin (75–150 mg/day). Aspirin use was documented as SP measure, in the case files of 100% (45/45) patients with CVD conditions. Among those who had CVDRFS alone, 11% (05/45) were prescribed aspirin as primary prevention measure and these five patients had two risk factors (hypertension and dyslipidemia). Patients (10%) without CVD/ CVDRFs did not receive aspirin.

**Table 1:** Risk category and percentage of Aspirin users

Risk category	n = 100	Aspirin users (%)	ADA recommendations for Aspirin use
Type 2 DM	100	50 (50/100)	-
CVD	45	100 (45/45)	Use for Secondary Prevention
Angina	31		
PVD	08		
MI	06		
CVDRFs alone	45		Use for Primary Prevention
One RF	32	00	
Two RFs	13	11% (5/45)*	
Hypertension*	35		
Dyslipidemia*	20		
Smoking	02		
Albuminuria	01		
Family history of CVD	01		
No CVD, No RF, age ≥ 40 years	10	00	Consider for Primary Prevention

* 11% Aspirin users for primary prophylaxis had two CVDRFs – hypertension and dyslipidemia

## Discussion

Aspirin prescription was documented in the case files of 50% of the diabetic patients. The captured data show encouraging results with reference to aspirin usage, which is 100% as secondary prevention strategy in established CVD conditions. The beneficial effects of aspirin in preventing subsequent cardiovascular events are well established.[3] Patient interviews and surveys report the prevalence of aspirin usage for secondary prevention of CVD events between the range of 37–82%.[4–9]

Forty-five percent of diabetic patients had CVDRFs alone and were potential candidates for aspirin primary prophylaxis. Among them, only 11% (05/45) who had two risk factors (hypertension and dyslipidemia) were prescribed aspirin and the remaining 89% (40/45) were deprived of this protective measure. According to ADA recommendations, age ≥ 40 years itself is a separate risk factor to be considered for aspirin primary prevention in DM patients, which if considered, even the remaining 10% patients with neither CVD nor CVDRFs ought to have received aspirin. The risk of CVD events among adults with diabetes is similar to that in nondiabetic individuals with CVD.[1–2] Research shows that low-dose aspirin is effective for primary prevention of CVD events in diabetic patients, who are at high risk.[3] Ever since (1997) the ADA recommended aspirin therapy for PP of CVD events in diabetic patients with high risk, the data published report the prevalence of self-reported use of aspirin for the same between the range of 5.7–59%.[4,6–9,10] Our observations(11%) are highly disappointing in this regard. Contraindications[3] for the use of aspirin were neither recorded in case files of patients not receiving aspirin nor were they prescribed any other antiplatelet drug. All those patients who ought to have received aspirin, but did not, were on more than three drugs. In an attempt to reduce the burden of poly pharmacy, physicians may give more preference to antidiabetic and antihypertensive drugs than to aspirin, in the absence of established CVD. Controversy about the cardiovascular benefits of aspirin in diabetics,[11] and concern about toxicities of aspirin on long-term usage might also have contributed to the under-prescription of aspirin.

## Conclusions

We evaluated the case files of a small number (100) of patients, being treated by restricted number of physicians belonging to two hospitals. Hence, the results cannot be generalized to the diabetic population. In the present study, we can only conclude that adherence to ADA Clinical Practice guidelines regarding aspirin use for primary prevention of CVD, is not satisfactory in the two teaching hospitals that we considered for our study. Hospital authorities and medical associations may play a major role in setting guidelines for the medical practitioners to encourage aspirin use for patients with diabetes.
